# Time trends in type 2 diabetes mellitus incidence across the BRICS from 1990 to 2019: an age-period-cohort analysis

**DOI:** 10.1186/s12889-021-12485-y

**Published:** 2022-01-11

**Authors:** Panglin Sun, Haoyu Wen, Xiaoxue Liu, Yudiyang Ma, Jie Jang, Chuanhua Yu

**Affiliations:** 1 Department of Epidemiology and Biostatistics, School of Public Health, Wuhan University, #115 Donghu Road, Wuhan, 430071 China; 2 Department of Global Health, School of Public Health, Wuhan University, Wuhan, Hubei China; 3 Global Health Institute, Wuhan University, #8 Donghu Road, Wuchang District, Wuhan, 430072 China

**Keywords:** Diabetes mellitus, Incidence, Age-period-cohort effect

## Abstract

**Background:**

The incidence rate of type 2 diabetes mellitus (T2DM) is rapidly increasing in Brazil, Russia, India, China, and South Africa (BRICS). The present study analyzed trends in T2DM incidence rate across the BRICS and associations with age, period, and birth cohort.

**Methods:**

The incidence rate was estimated by the data obtained from GBD 2019 (Global Burden of Disease Study 2019) and was analyzed with the age-period-cohort framework. Incidence rates of T2DM (1990–2019) were collected for each 5-year age group (from 25 to 29 to 85–89 age group) stratified by gender from the Global Burden of Disease 2019 Study.

**Results:**

In 2019, the the incidence rate of T2DM was 280.2 per 100,000 across the BRICS. Between 1990 and 2019, the incidence rate of T2DM among the BRICS population increased by 83.3%. In each period, as age increases, the incidence rate of T2DM in China and Russia first increased and then decreased, while the incidence rate of T2DM in Brazil, India and South Africa first increased and then decreased slightly with age group. Deteriorating period and cohort risks for incidence rate of T2DM were generally found across the BRICS.

**Conclusions:**

The number of diabetic patients in the BRICS countries has continued to increase and the growth rate has been stable in the past 30 years, which is dependent on age and some other environmental factors. Some possible factors influencing T2DM incidence are analyzed and hypotheses generated through the age and period effects.

**Supplementary Information:**

The online version contains supplementary material available at 10.1186/s12889-021-12485-y.

## Background

In 2010, about 12.9 million people were killed by ischemic heart disease and stroke, an increasingly important risk factor for which is diabetes [1, 2]. Diabetes mellitus is a chronic metabolic non-communicable disease (NCD), which has spread globally. The World Health Organization (WHO) has identified diabetes mellitus as one of the four main NCDs meriting close attention [3], as the number of deaths due to diabetes increased by 31.1% between 2006 and 2016 globally. As of 2015, more than 415 million adults have diabetes, and this number is expected to increase to 642 million by 2040.

Analyzing the incidence trend of T2DM can help to understand and control the burden of disease. Brazil, Russia, India, China, and South Africa constitute the political and economic blocs of countries with rapid economic development, with nearly half of the world’s population [4]. These five countries include several different races, have different cultures and living habits, and have all experienced medical care and socio-economic development. Moreover, several of these countries are experiencing aging, and aging likely drives a continued increase in the incidence and mortality of diabetes [5]. Statistical analysis of these countries can help formulate public health policies and estimate and analyze future trends.

Aging likely drives a continued increase in the incidence and mortality of diabetes. Timely population-level interventions aiming for obesity prevention, healthy diet and regular physical activity should be conducted, especially for men and earlier birth cohorts at high risk of diabetes.

This study aims to examine time trends from 1990 to 2019 and the age, period, and cohort effects on T2DM incidence. Using data derived from the Global Burden of Disease Study 2019, we provide a first comprehensive report of the striking differences in T2DM across Brazil, Russia, India, China, and South Africa, as well as associations with age, period, and birth cohort, over the past 30 years.

## Methods

### Data sources

The population data of BRICS and the number of new T2DM in the population of BRICS were obtained from the GBD 2019 study. GBD 2019 study not only provides population estimates in 204 countries and territories from 1950 to 2019 [6], but also provides a comprehensive assessment of incidence, prevalence, and years lived with disability (YLDs) for 369 diseases and injuries in 204 countries and territories from 1990 to 2019 [6]. The original data, which GBD adapted to estimate population, was mainly from UNSD [7], UN Population Division [8], UN DYB [9], the Integrated Public Use Microdata Series (IPUMS) [10], and the Population Research Center at The University of Texas at Austin [11].

The subject of this study is T2DM, and the criteria for T2DM are detailed in Additional file 1. Considering that GBD’s data estimation processing for T2DM also involves overall Diabetes Mellitus and Diabetes Mellitus Type 1, the criteria are also attached in Additional file 1. Conceptually, the work of data estimation is divided into the following steps: (1) compiling data sources through data identification and extraction. (2) data adjustment; (3) estimation of incidence by using DisMod-MR 2.1 [12]. With regard to access to data sources, it is important to emphasize that only 20% of Diabetes Mellitus estimates are available by type, and the diagnostic criteria in the methodological sections are not sufficiently specific [12]. Therefore, the estimate for T2DM is actually derived from the estimate of overall Diabetes Mellitus minus the estimate of Diabetes Mellitus Type 1 for each age, sex, and location from 1990 to 2019.

All the data mentioned above can be obtained here: http://ghdx.healthdata.org/GBD-resultstool, contains core summary results for GBD 2019.

### Statistical analyses

The present study used the Age-Period-Cohort Model to analyze the data. APC model is a generalized linear model that takes age, period and cohort as independent variables, takes the occurrence of a certain observed event or a phenomenon in a population as the dependent variable, and assumes that the dependent variable obeys a certain probability distribution. This model is based on retrospective repeated measurement cross-sectional data (age-specific incidence rate in different periods), fits incidence rate, and separates age effects, period effects, and cohort effects, so as to quantify the influence of age, time and birth cohort factors on disease rate.

To conduct APC analysis, the incidence rate of T2DM in a specific age group and time period is displayed in the form of a two-dimensional contingency table (Additional file 2 and 3). In order to keep the number of APC model parameters at a manageable level and reasonably obtain a smooth curve of the time-effect curve, we divided age-specific incidence rate into groups of 5 years (25 to 29, 30 to 34… 85 to 89). Because the occurrence of incidence from T2DM in those aged< 25 years is rare, they were not considered in present study. The APC model requires that age and period must be divided at equal intervals. Therefore, period data is divided into groups of 5 years (1990 to 1994, 1995 to 1999…2015 to 2019). Since the birth cohort is defined by the age of the subject and the date of the incident, that is, cohort = period-age, the corresponding birth cohort is 1903 to 1907 (median, 1905) to 1988 to 1992 (median, 1990). When the age interval and the period interval are equal in width, the diagonal line of the age-period-specific T2DM incidence rate two-dimensional contingency table represents the birth cohort. The age groups form the rows of the Additional file 2 and Additional file 3 and period forms the columns, the diagonal line from the lower left corner to the lower right represents the birth cohort. The age effects represent a differing risk of the outcome associated with different age brackets; the period effects represent variations in the outcome over time that influence all age groups simultaneously; the cohort effects are associated with changes of the outcome across groups of individuals with the same birth years. Net drift represents the overall log-linear trend by period and birth cohort and indicates the overall annual percentage change of the expected age-adjusted rates over time; local drift represents the log-linear trend by period and birth cohort for each age group and indicates the annual percentage change of the expected age-specific rates over time; the longitudinal age curve indicates the expected age-specific rate in a reference cohort adjusted for period effects [13].

The estimated parameters were obtained by the age-period-cohort Web Tool provided by the National Cancer Institute [14]. Wald chi-square tests were adopted for the significance of the estimable parameters and functions.

## Results

### Trends in T2DM incidence

**Fig. 1:**
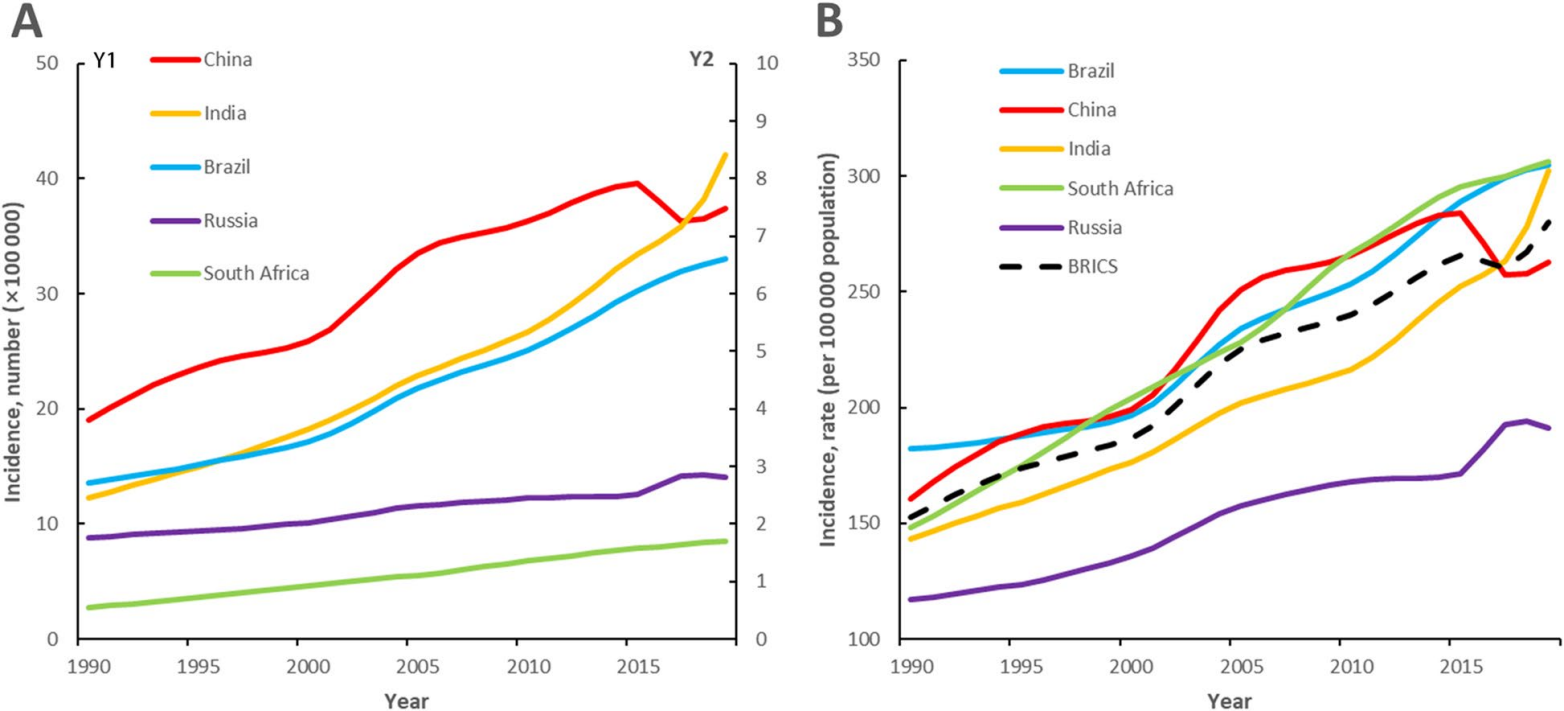
Number new of T2DM, and incidence rate of T2DM across Brazil, China, India, Russian Federation, and South Africa between 1990 and 2019. Note: (A) Y2 axis for Brazil, Russia, and South Africa

Table 1 and Fig. 1 show the trend of incidence of T2DM in BRICS. In 2019, there were 192.58 million patients with T2DM in BRICS. From 1990 to 2019, the incidence rate of type 2 diabetes in BRICS rose from 152.9 per 100,000 to 280.2 per 100,000, with China having the lowest growth rate of 63.6%, and India having the highest growth rate of 110.9%. In 2019, the incidence rate of T2DM in Russia was at the lowest level among BRICS (191.3 per 100,000). The other three countries: Brazil, India, and South Africa have similar incidence rate of type 2 diabetes, all exceeding 300 per 100,000.

**Table 1:** Characteristics of T2DM incidence in BRICS Countries Between 1990 and 2019 BRICS: Brazil, Russia, India, China, South Africa; T2DM: Type II diabetes mellitus

		BRICS		Brazil		Russia		India		China		South Africa	
		1990	2019	1990	2019	1990	2019	1990	2019	1990	2019	1990	2019
Population	Total, n × 1,000,000	2376.0	3232.	148.8 (138, 159)	216.7 (190, 243)	151.0 (139, 163)	146.7 (129, 165)	855.6 (792, 919)	1390.7 (1238,1559)	1183.7 (1103, 1272)	1422.4 (1239, 1597)	36.8 (33, 41)	55.6 (49, 63)
	Percentage of global	44.4	41.8	2.8	2.8	2.8	1.8	16.0	18.0	22.1	18.4	0.7	0.7
T2DM	Incidence rate per 100,000	152.9	280.2	182.2	304.6	116.9	191.3	143.4	302.5	160.7	262.9	148.3	306.1

Note: The data corresponding to the BRICS data bar is the sum of the Brazil, Russia, India, China, and South Africa data

Abbreviations: *T2DM* Type 2 diabetes mellitus

*BRICS* Brazil, Russia, India, China, South Africa

Additional file 2 and Additional file 3 show the number new of T2DM and incidence rate of T2DM by age, period, and median birth cohort in BRICS. From 1990 to 2019, the incidence of T2DM in the BRICS countries has generally increased. From 1990 to 2015, the incidence rate of T2DM in China, Brazil, India, and South Africa all increased significantly, and the incidence rate of T2DM in Russia increased more slowly than the other four countries. From 2001 to 2005, the incidence rate of T2DM in China experienced a five-year rapid increase, with an increase of 22.1% within five years (from 205.6 per 100,000 in 2001 to 251.0 per 100,000 in 2005). From 2015 to 2019, the incidence rate of T2DM in India increased rapidly, with an increase of 19.8% (from 252.6 per 100,000 in 2015 to 302.5 per 100,000 in 2019); The incidence rate of T2DM in Russia has also experienced three years of rapid growth during this period, from 171.2 per 100,000 in 2015 to 192.8 per 100,000 in 2017, and then the growth rate has declined after 2018 (194.0/100000 people), the incidence rate dropped to 191.3 per 100,000 people in 2019; During the same period, the incidence rate of T2DM in China first experienced a three-year negative growth, from 284.0 per 100,000 in 2015 to 257.4 per 100,000 in 2017 (a decrease of 9.4%), and then a steady increase to 262.9 per 100,000 in 2019.

### Age-specific incidence rates for T2DM

**Fig. 2:**
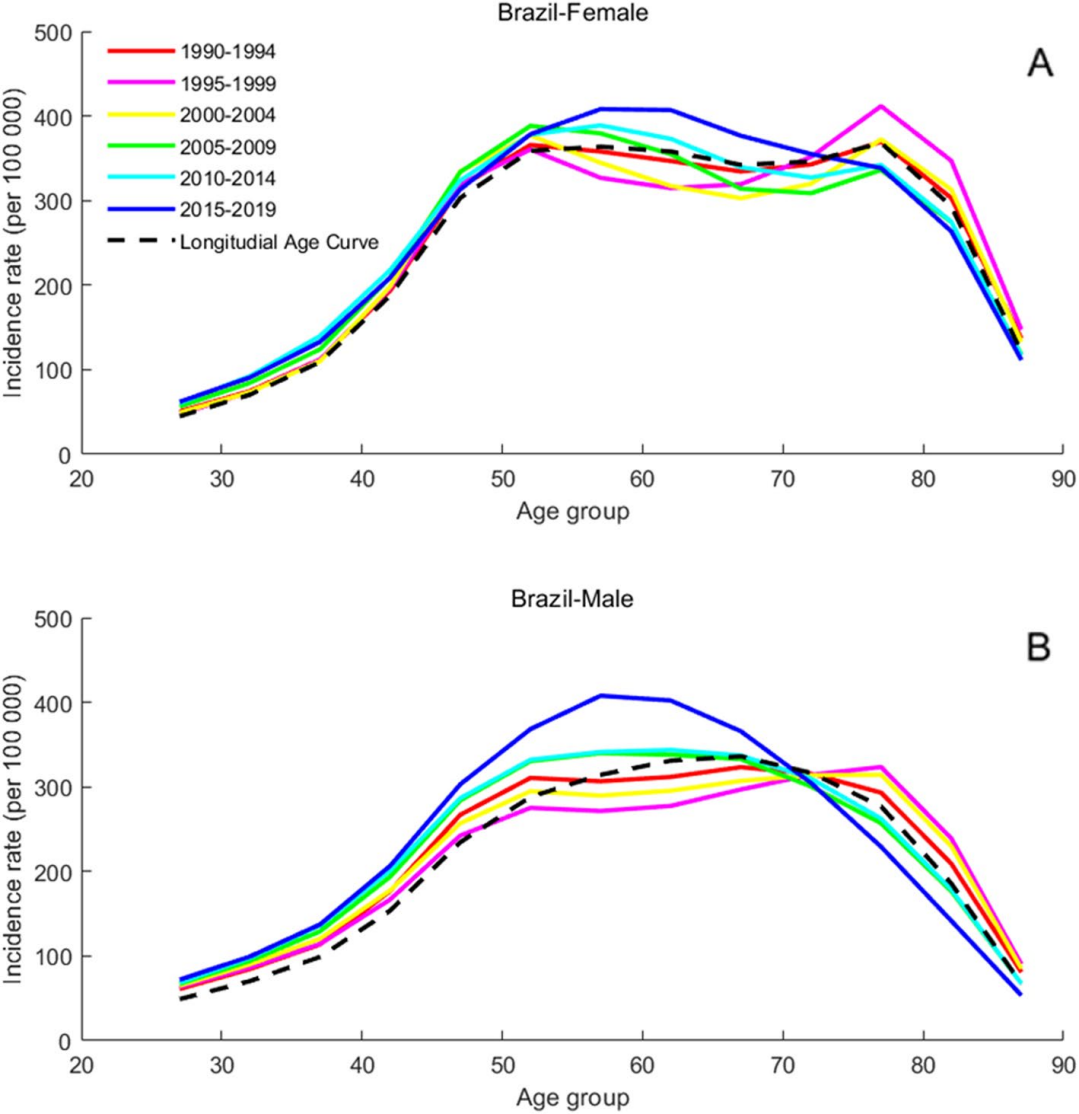
Age-specific incidence rates of T2DM by period in Brazil between 1990 and 2019. Note: Survey years were arranged into consecutive 5-year periods from 1990 to 1994 (median, 1992), 1995 to 1999 (median, 1997), 2000 to 2004 (median, 2002), 2005 to 2009 (median, 2007), and 2010 to 2014 (median, 2012), and 2015 to 2019 (median, 2017). Longitudinal age curves were estimated by age-period-cohort model and indicated the expected age-specific rate of T2DM incidence. The age-specific curve for women is shown in the A-block of each graph, while the age-specific curve for men is shown in the B-block

**Fig. 3:**
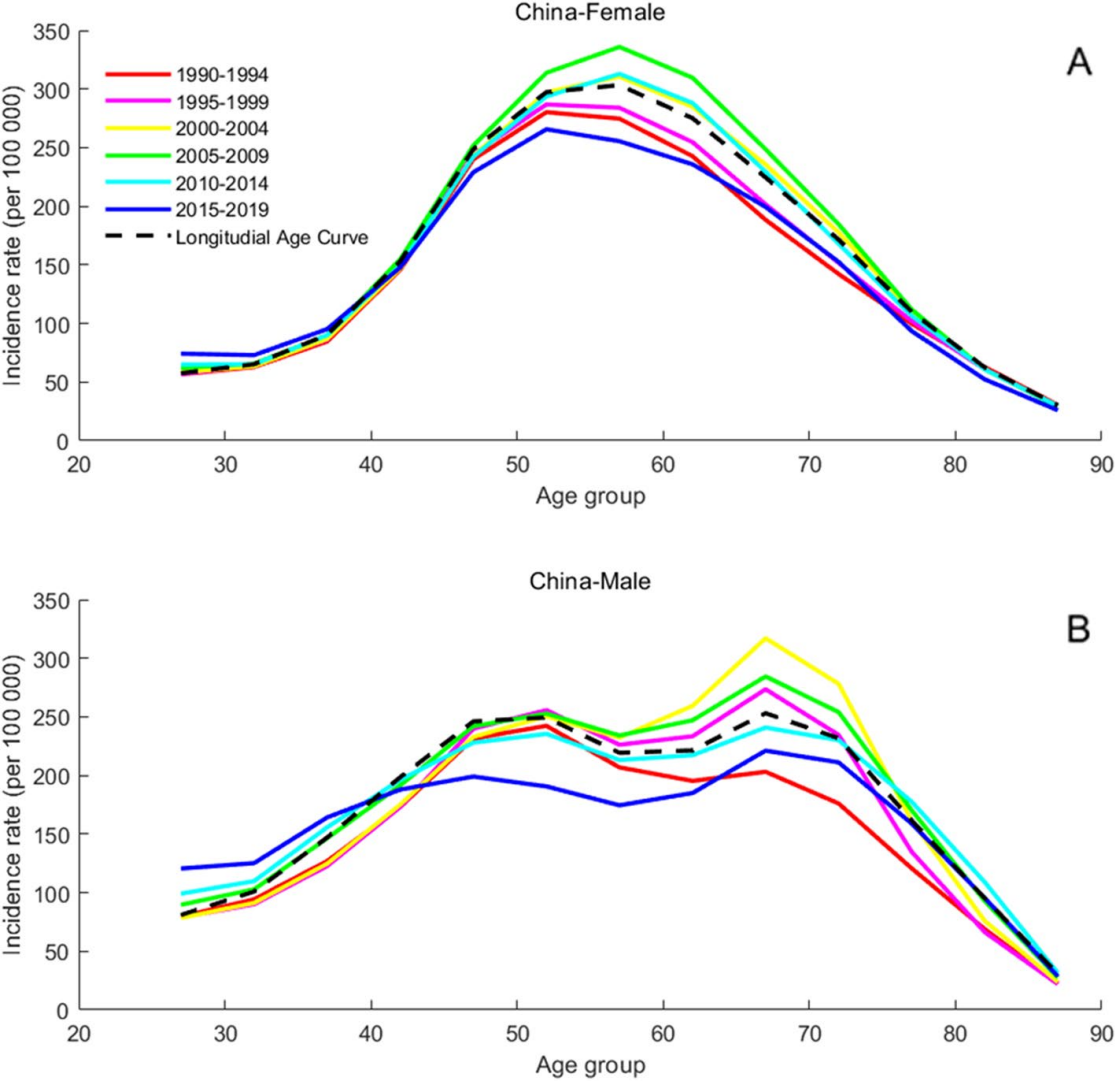
Age-specific incidence rates of T2DM by period in China between 1990 and 2019. Note: Consistent with the note in Fig. 2

**Fig. 4:**
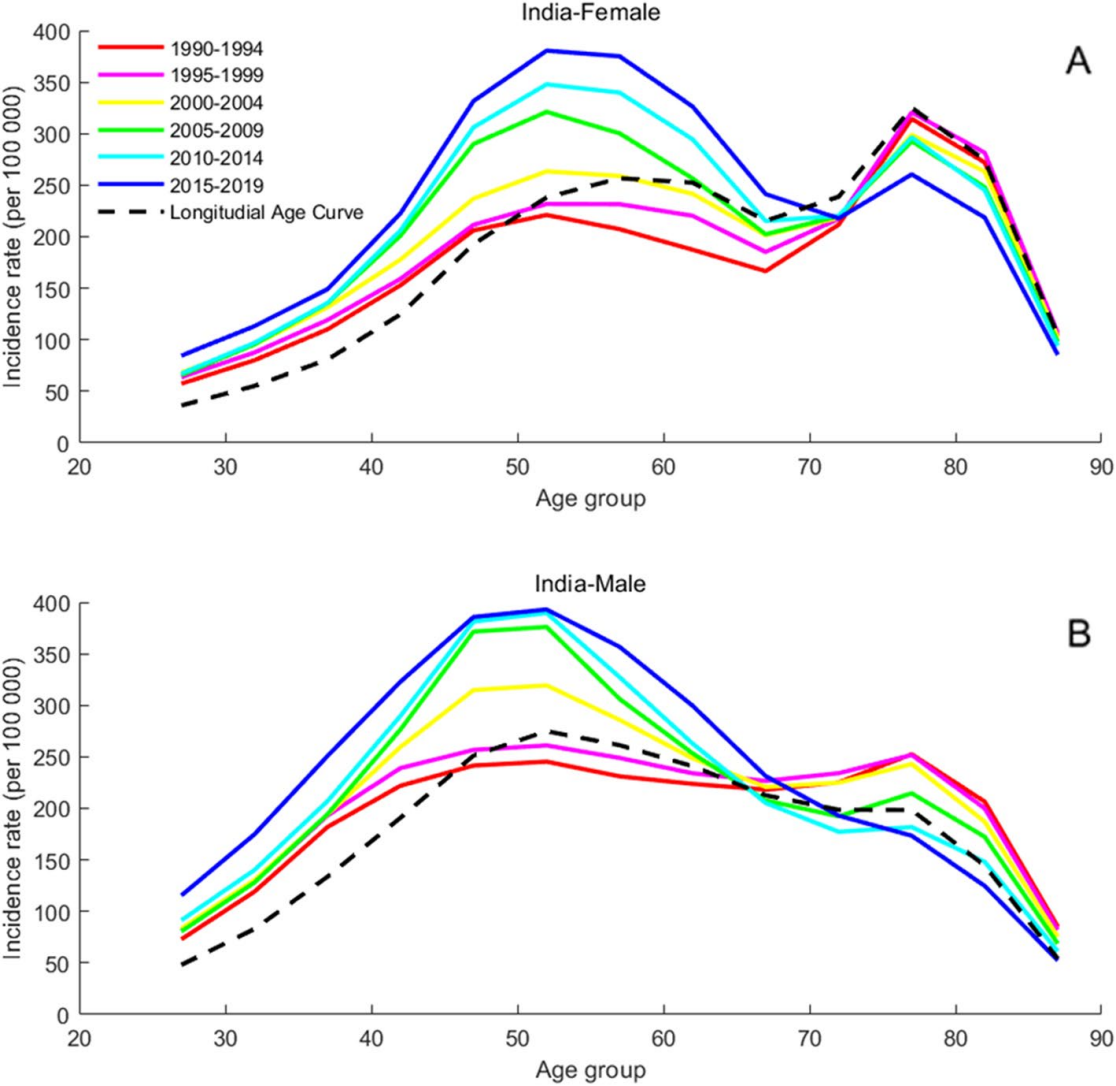
Age-specific incidence rates of T2DM by period in India between 1990 and 2019. Note: Consistent with the note in Fig. 2

**Fig. 5:**
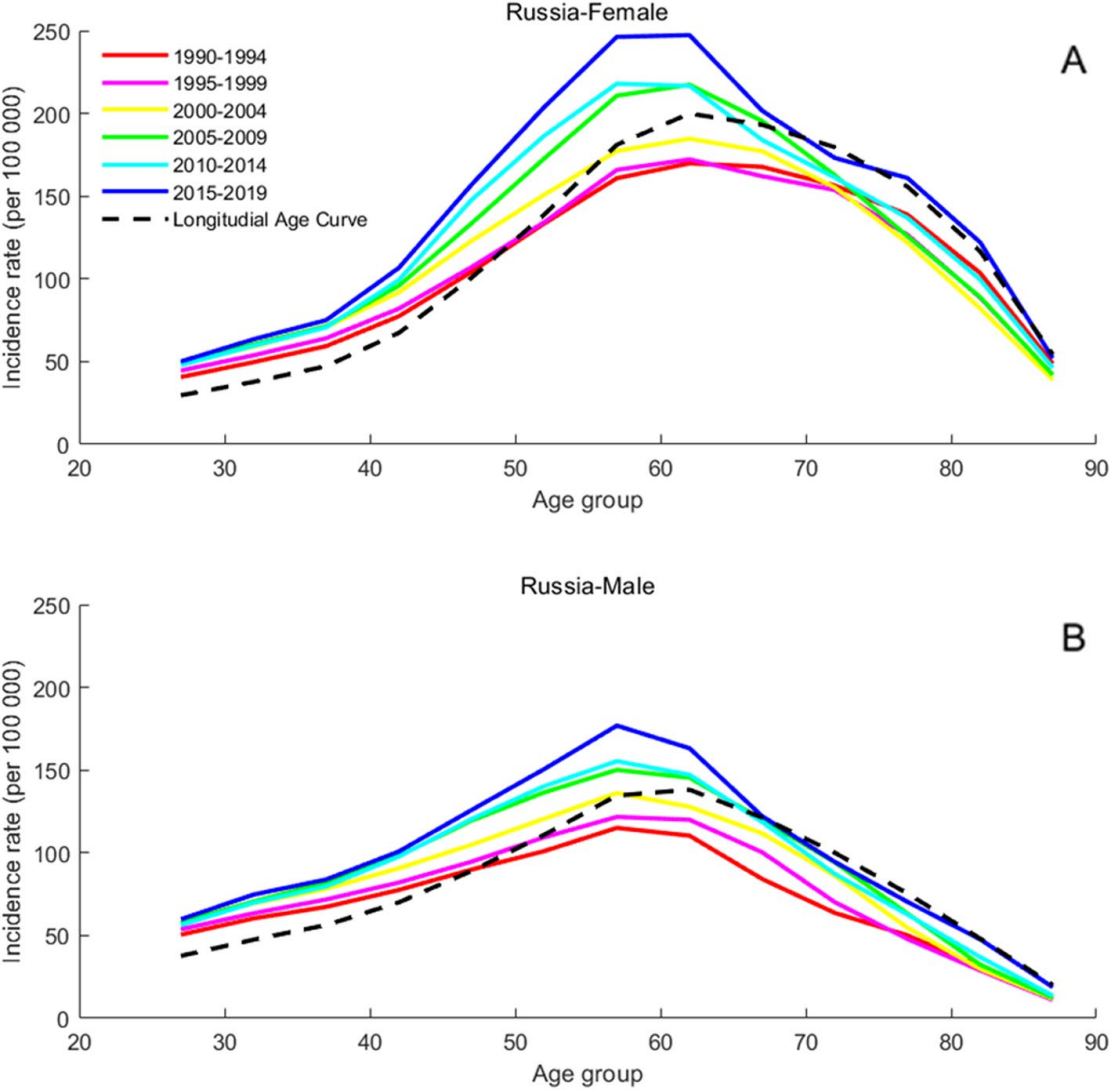
Age-specific incidence rates of T2DM by period in Russia between 1990 and 2019. Note: Consistent with the note in Fig. 2

**Fig. 6:**
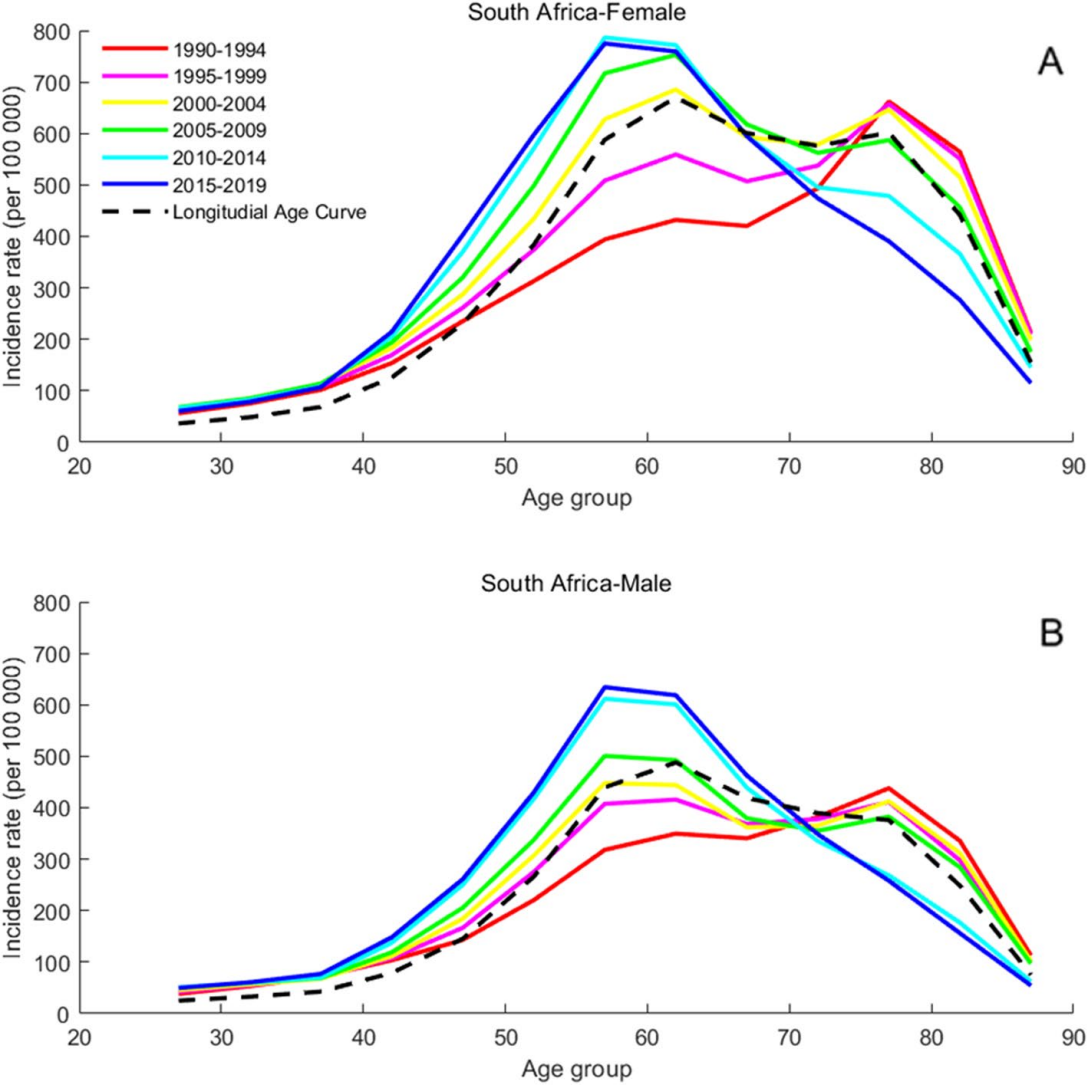
Age-specific incidence rates of T2DM by period in South Africa between 1990 and 2019. Note: Consistent with the note in Fig. 2

The present study arranged the incidence of T2DM into consecutive 5-year periods from 1990 to 1994 (median, 1992) to 2015 to 2019 (median, 2017) and 19 consecutive cohorts, including those born from 1903 to 1907 (median, 1905) to 1988 to 1992 (median, 1990). Figures 2, 3, 4, 5 and 6 show trends in T2DM incidence rate across the BRICS countries from 1990 to 2019. Figure 4 shows that the incidence rate trend of T2DM in Indian women is roughly the same as that of men. For Russia and South Africa, a similar conclusion can be drawn from Fig. 5 and Fig. 6. As can be seen from Fig. 2A, there are two peaks on the curve, which correspond to the age groups 50 to 60 and 75 to 80. However, in Fig. 2B, the incidence rate among Brazilian men aged 75 to 80 is not an extreme point (except for the “1995–1999” curve). The difference between men and women is also found in China, as can be seen from Fig. 3.

### Net drift and local drift in different age groups

**Fig. 7:**
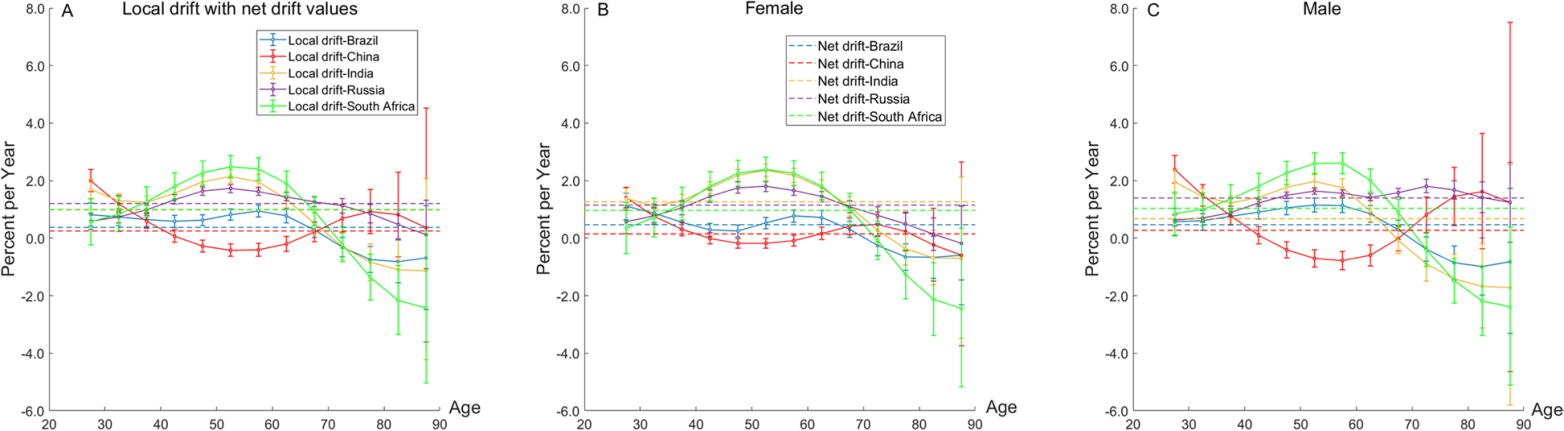
Local drift with net drift values for T2DM incidence rate in Brazil, China, India, Russia, South Africa from 1990 to 2019. Note: **A**-**C** use the same set of legends; **B** and **C** are the result of data on women and men, respectively

Net drift indicates the overall annual percentage change across the whole study period, whereas local drift indicates the annual percentage changes in incidence rate for each age group relative to the net drift (Fig. 7). The overall net drift was similar in Brazil (0.37% [95%CI, 0.21 to 0.53]) and China (0.25% [95%CI, − 0.01 to 0.51]). The overall net drift was similar in India (0.98% [95%CI, 0.76 to 1.21]), Russia (1.21% [95%CI, 1.10 to 1.31]), and South Africa (1.00% [95%CI, 0.71 to 1.28]). This fact reflects that the overall increase in incidence rate in India, Russia, and South Africa was higher than that in Brazil and China across the study period. There were marked sex differences in the overall annual change in India, with more deterioration in incidence rate in women than in men (1.26%[95%CI, 1.06 to 1.47] versus 0.68%[95%CI, 0.38 to 0.97], respectively).

Local drift reflects additional age variations in incidence rate trends. For example, for the Chinese women 25 to 39 years of age, local drift values are higher than 0, indicating deterioration in T2DM incidence. The exception were people 45 to 65 years of age in China (− 0.42% to − 0.21%), people 75 to 89 years of age in Brazil (− 0.81% to − 0.32%), India (− 1.13% to − 0.29%), and South Africa (− 2.42% to − 0.22%). The worst deterioration was for men 55 to 59 years of age in South Africa (2.60%/y) and women 50 to 54 years of age in South Africa (2.39%/y), with the similar situation for women of the same age in India (2.36%/y).

The local drift curves of Brazil and China first showed a decrease and then an increase across the age, with India, Russia, and South Africa showed an opposite trend.

### Age-period-cohort effects on T2DM incidence rate

**Fig. 8:**
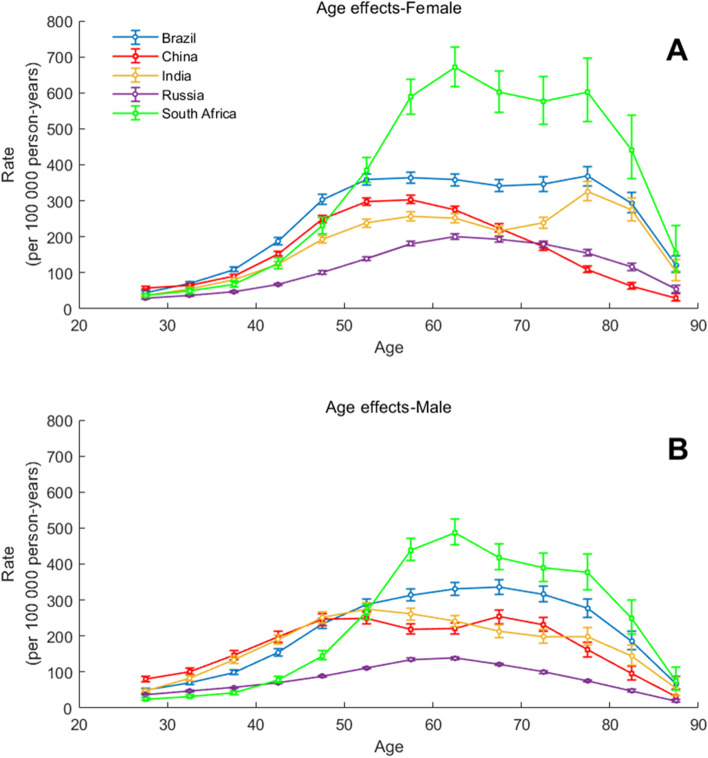
Parameter estimates of age effects on T2DM incidence rate in Brazil, Russia, India, China, and South Africa from 1990 to 2019. Note: All the data were analyzed by gender, with block A corresponding to female and block B corresponding to male

**Fig. 9:**
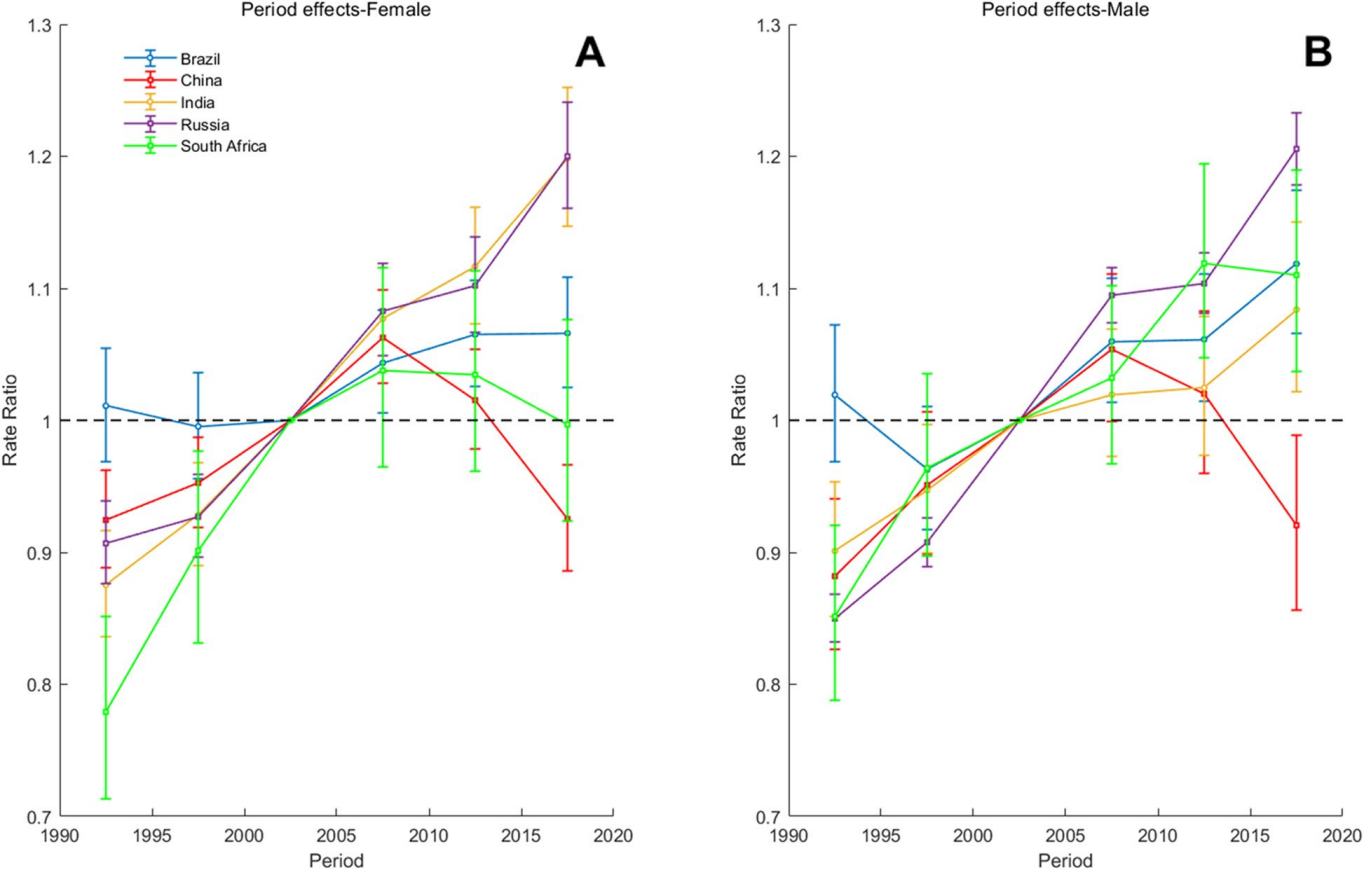
Parameter estimates of period effects on T2DM incidence rate in Brazil, Russia, India, China, and South Africa from 1990 to 2019. Note: Consistent with the note in Fig. 8

**Fig. 10:**
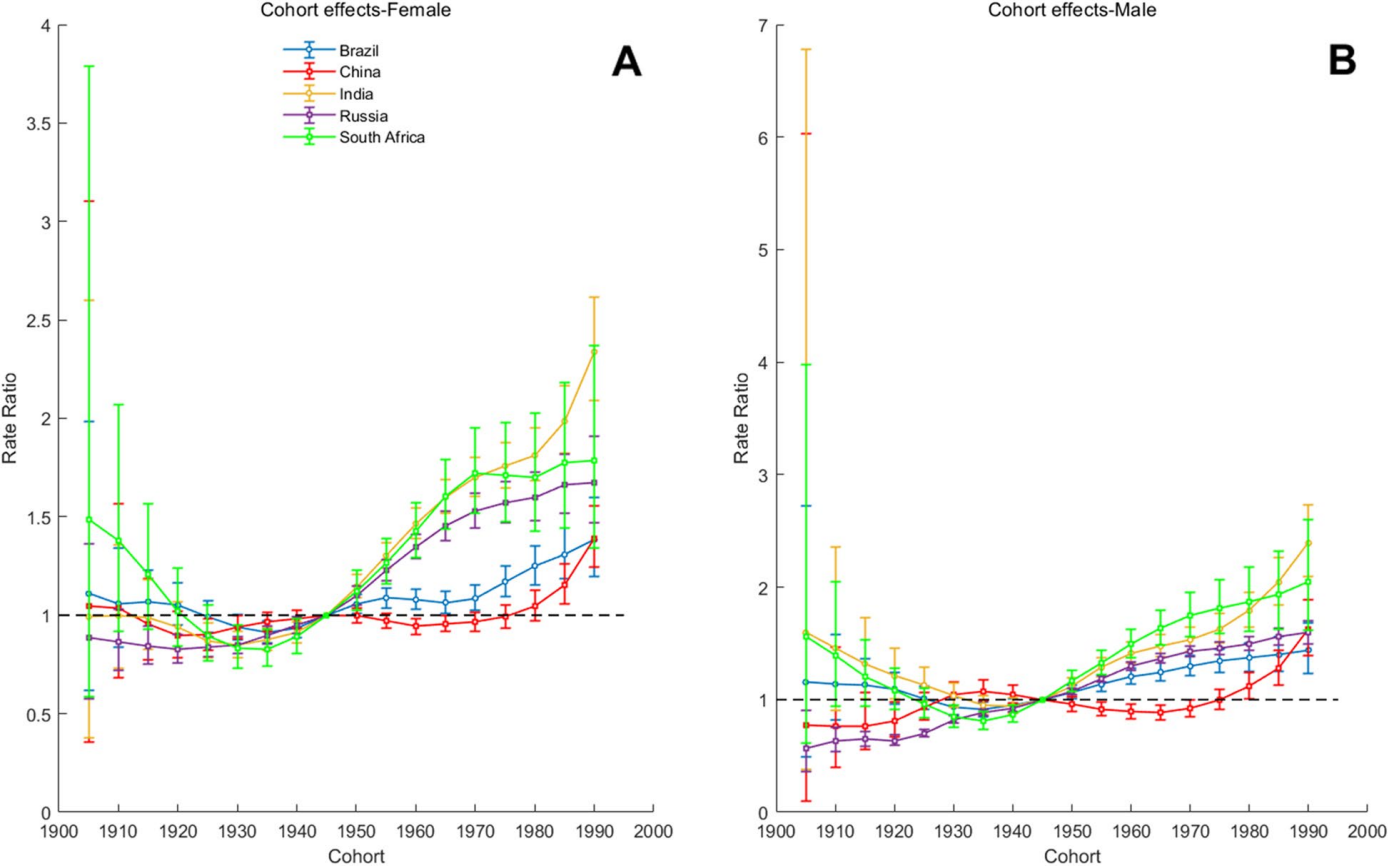
Parameter estimates of cohort effects on T2DM incidence rate in Brazil, Russia, India, China, and South Africa from 1990 to 2019. Note: Consistent with the note in Fig. 8

Figures 8, 9 and 10 show the estimated effects of age, period, and cohort on the T2DM incidence rate. Tables 2 and 3 shows the fitting formula for the longitudinal age trend of incidence-age in the BRICS countries, while Table 2 corresponds to women and Table 3 corresponds to men. Accordingly, the goodness of fit of each curve is also given in the table. The incidence rate of T2DM in South Africa increases fastest with age. Brazil, China, Russia, and South Africa all have a rapid decline in T2DM incidence rate in the later years of life. From the curve equation and Fig. 8, it is not difficult to find that middle-aged people are more likely to develop T2DM. The value of *RR*_*age* _ *group* _ *x*_ can also be known from the data, which indicates the relative risk for T2DM incidence rate in age group x compared with age group 25–29. For example, *RR*_60 − 64_ were 7.966 for women in Brazil, 4.78 for women in China, 18.28 for women in South Africa.

**Table 2:** Estimated longitudinal age trend for women

Location	Curve Equation	R square
	$$\mathrm{Incidence}\ \mathrm{rate}=a\ast {e}^{-{\left(\frac{mean\_ age-b}{c}\right)}^2}$$	
Brazil	*a* = 3.955 ∗ 10^−3^; *b* = 64.51; *c* = 25.90	0.9045
China	*a* = 3.098 ∗ 10^−3^; *b* = 57.29; *c* = 18.77	0.9798
India	*a* = 2.756 ∗ 10^−3^; *b* = 67.72; *c* = 29.46	0.7953
Russia	*a* = 2.041 ∗ 10^−3^; *b* = 64.79; *c* = 21.24	0.9705
South Africa	*a* = 6.744 ∗ 10^−3^; *b* = 67.24; *c* = 20.03	0.9540

Note: *b* indicates the age when the incidence rate in the positive fit curve peaks

**Table 3:** Estimated longitudinal age trend for men

Location	Curve Equation	R square
	$$\mathrm{Incidence}\ \mathrm{rate}=a\ast {e}^{-{\left(\frac{mean\_ age-b}{c}\right)}^2}$$	
Brazil	*a* = 3.502 ∗ 10^−3^; *b* = 63.31; *c* = 23.38	0.9625
China	*a* = 2.588 ∗ 10^−3^; *b* = 57.33; *c* = 26.83	0.8832
India	*a* = 2.697 ∗ 10^−3^; *b* = 58.48; *c* = 26.13	0.9171
Russia	*a* = 1.377 ∗ 10^−3^; *b* = 60.83; *c* = 21.22	0.9053
South Africa	*a* = 4.791 ∗ 10^−3^; *b* = 65.92; *c* = 18.37	0.9569

Note: Consistent with the note in Fig. 2

As can be seen from Fig. 9, the period has similar effect on the T2DM incidence rate in Brazil, India, Russia and South Africa: the rate ratio value of these countries is greater than 1, suggesting no improvements for the whole population in the past decade. The effect in the period 2015 to 2019 had a positive impact on T2DM incidence in China. By comparing Fig. 9A and Fig. 9B, it can also be seen that the negative impact of period effects on South African women is slowly being controlled after 2010. The period range for 2015–2019 corresponds to the rate ratio: 0.9969, which is less than 1.

In recent years, the cohort effect has adversely affected the incidence rate of T2DM in the population of all BRICS countries. Brazil is less affected by cohort, while India and South Africa are the ones most affected by the cohort, and the incidence rate of births in India and South Africa has been increasing gradually since 1935. For China and Russia, the cohort effect has a beneficial effect on the incidence rate of people born earlier.

## Discussion

Although the incidence of T2DM in the Chinese and Russian populations has experienced a period of decline in the past five years, the data analysis of the past 30 years shows that this indicator has increased significantly in the BRICS countries. In India the incidence rate has increased by 110.9% (143.44 to 302.5) compared with 30 years ago; even in China, where the incidence rate has increased the least, the incidence rate has increased by 63.6% (160.72 to 262.5) in the past 30 years. The present study found that among people under 49, the incidence rate of T2DM from all over the world increased with age; and the incidence rate of T2DM in people aged 50–79 was significantly higher than that in other age groups. Through APC model, we can know the effect of age, period, and cohort on the incidence of T2DM in various countries (Fig. 5). Period effects are generally related to the social and economic environment. Cohort effects represent changes across groups of individuals born in the same years [5].

For China, age was a risk factor for T2DM. The incidence rate of T2DM in people aged 55–59 and 60–64 is more than 5 times that of 25–29 years old. In Fig. 10A and B, the curve representing China has a trough at 1960–1964, which may be explained by the 1959–1961 famine in China. As can be learned from existing studies, fetal and early childhood exposure to famine was associated with obesity risk in adulthood, which may further increase the risk of diabetes, including cardiovascular disease [15, 16]. According to China Noncommunicable Disease Surveillance 2010, location was a significant association with diabetes. Compared with rural areas, diabetes medications are more commonly used in urban areas. Approximately two-fifths of diabetic patients in urban areas, but less than one-quarter of diabetic patients in rural areas, were aware of their diagnosis [17]. The lack of relevant professionals has led to a very low level of integrative management in China, a relatively complete diabetes integrative management system is only deployed in a few large-scale hospitals [18]. A recent nationwide survey in China, reported that only one-third of the treated diabetes cases had achieved adequate glycemic control, as opposed to three-quarters in the United States [19]. The number of new T2DM patients in China each year continued to increase until 2015, and it is higher than the other four BRICS countries. The imperfection of the public medical and health system may be a reason for the gradual increase in the effect rate ratio between 1990 and 2010 and the cohort effect rate ratio after 1980. Further risks relate to greater body mass index and a larger waist circumference, with other significant associations being male gender, a family history of diabetes, and increased systolic blood pressure, low-density lipoprotein–cholesterol, and triglyceride levels [20]. Both cigarette and alcohol use were associated with a lower risk of diabetes [21]. Since 2011, the Chinese government has begun to implement a series of response measures to improve public health. For example, China’s 2030 Sustainable Development Goals that include reducing noncommunicable disease mortality by one-third and monitoring the changes over time. This series of measures may reflect the period effect rate ratio between 2010 and 2019 and the cohort effect rate ratio before 1980. Nevertheless, the rapid economic growth in recent decades brings about changes in lifestyle which included increased high-calorie, high fat, high-sugar, and high-sodium diets and decreased physical activity, all these suggest that the current situation is not optimistic [21].

The incidence rate of T2DM in India also has a significant unfavorable trend, especially for people over 25 years of age. According to International Diabetes Federation (IDF), Diabetes Atlas (2015), 8.8% of adults between the ages of 20 and 79 have been diagnosed with diabetes, approximately 415 million. The period and cohort effects have both risen sharply in 30 years. An existing study shows that unhealthy diet, obesity, lack of exercise, serum triglycerides and low HDL cholesterol can be used to explain 80% of the attributable risk of diabetes in the Indian population [20, 22]. The increase in the incidence of T2DM is also affected by various behaviors under modern lifestyles, such as exposure to industrial chemicals, smoking, lack of sleep, and depression [23–25].

In South Africa, the cohort and period effects have grown similarly to India in the past 30 years. The incidence rate of T2DM in South Africa increased from 148 (per 100,000) in 1990 to 306 (per 100,000) in 2019. As mentioned in the previous discussion, diabetes is well known to be associated with urban/rural location [26–29]. Compared with people living in rural areas, T2DM has a greater impact on the lives of people living in urban areas [30]. The risk factors for diabetes mellitus in the South Africa include age, smoking, HIV status and community [31]. Particularly, the *RR*_60 − 64_ were 18.28 for women in South Africa, which means the risk of T2DM incidence rate in women aged 60 to 64 years is more than 18 times that of women aged 25 to 29 years old. According to further results obtained from other studies, of those with diabetes, individuals who are more likely to have undiagnosed diabetes are those who are older, from a lower household socio-economic position and with a lower level of education. In addition, there was strong evidence that BMI was associated with diabetes. An existing study shows that the highest risk groups were those of older age and those with obesity [31].

Brazil has been experiencing a gradual increase in the prevalence of T2DM over the last 3 decades, as well as an increase in the prevalence of important risk factors, such as obesity. The prevalence of DM in Brazil is estimated to be a long-term upward trend, and international estimates believe that it will increase from 6 to 7.8% in 2030 [32]. An existing study shows that the rates of overweight (BMI > 25 kg/ m^2^) and obesity are spreading among study subjects of all age groups, genders and all income levels in Brazil, especially low-income families [33]. Specifically, over the past 36 years, the excessive weight (BMI .25 kg/ m^2^) has risen from 16 to 50% in men and from 28 to 48% in women, as seen in successive data from the Household Budget Survey [34]. And over the past 6 years, the prevalence of obesity (BMI .30 kg/ m^2^) has risen from 8.9 to 12.5% in men and from 13.1 to 16.9% in women [35, 36]. Furthermore, according to the conclusions of existing epidemiological studies, physical activity may bring a range of health benefits, such as slow the occurrence and progression of insulin resistance and improve blood sugar control, blood pressure and lipid characteristics [35, 36]. In Brazil, however, an estimated 41% of adults are not sufficiently active. so based on the above data, unhealthy lifestyle such as physical inactivity may be considered important variables for T2DM and CVD [37].

Russia had an increasing number of T2DM patients from 1990 to 2019. The incidence rate of T2DM in Russia increased from 116.88 (per 100,000) in 1990 to 191.26 (per 100,000) in 2019. From 1990 to 2019, the period effect has been on the rise, and faster than in the other four countries, high rates of smoking and obesity may explain this phenomenon. According to a study of Middle East, Africa and Russia, obesity is the most critical risk factor of T2DM [38]. Further risks relate to increasing urbanization, as people in urban areas are more exposed to air pollution [39]. In addition, BMI and age were both significantly higher in rural areas versus urban areas [40], which may lead to differences in T2DM prevalence in different regions, further affecting risk factors attributed to urbanization [41].

To some extent, the BRICS are in the different stages of health transition, which is reflected in the different trends of each country. As mentioned in the previous discussion, some of China’s social policies may have had a positive impact on the construction of public health. There is a similar example in Brazil, various factors such as the stable development of society brought about by economic growth after 1994, the universal health care movement from the 1970s to the 1980s, and the establishment of BUHS, which provided free essential medicines for diabetes control in 1990 [42]. In contrast, Russia and India have not done enough in this regard, for example, their lack of effective pre-screening for diabetes, which makes it more difficult for clinicians to control diabetes, and it is worth noting that Russia has not even established a gestational diabetes detection system [43]. The active response of the health system and the construction of social health consensus can effectively reduce the incidence and prevalence of T2DM.

There are some limitations in this study. First, the data used in this study comes from a single source, all derived from the research results of GBD2019. This secondary data does not fully reflect certain trends. Second, the statistics object does not include the data of people under 25 years old. This is due to a priori knowledge that T2DM occurs mostly in middle-aged people, while ignoring the current situation that the incidence of T2DM among adolescents worldwide has become more and more intense in recent years. Based on existing research for Chinese groups, a conspicuous association between famine exposure during the early-childhood period and an increased risk of hyperglycemia in adult women [44]. Third, although period and cohort effects were estimated in this study, our age-period-cohort analysis was based on the estimated cross-sectional data of GBD from 1990 to 2019, which was not a cohort study. Large cohort studies in different countries are needed to establish location- and time-specific relative risks.

## Conclusion

The incidence rate of T2DM in the BRICS countries has been continuously rising for 30 years. This trend has not been curbed, although the incidence rate of T2DM in middle-aged and older persons has decreased in recent years compared to 10 years ago, the risk of T2DM has increased in the younger population. In addition, we used the APC model to calculate the incidence over the past 30 years, and obtained age-period-cohort effect equivalents in these five countries. Overall, the risk of T2DM in middle-aged people is significantly higher than in younger and older people, and in the population from 2005 to 2019 it is higher than in 1990–2005, and the birth queue after 1945 is more likely to develop T2DM.

## Supplementary Information

**Additional file 1.** The criteria for overall Diabetes Mellitus, Diabetes Mellitus Type 1 and Diabetes mellitus Type 2.

**Additional file 2.** Title: Number new of T2DM by age, period and median birth cohorts in BRICS,1990 to 2019. Description: The file includes five worksheets: ‘Brazil-Female’, ‘Brazil-Male’, ‘China-Female’, ‘China-Male’, ‘India-Female’, ‘India-Male’, ‘Russian Federation-Female’, ‘Russian Federation-Male’, ‘South Africa-Female’, ‘South Africa-Male’. In each sheet, the data indicate the number of new T2DM female/male patients in each specific age group during the period. The diagonal line from the lower left corner to the lower right represents the birth cohort.

**Additional file 3.** Title: Incidence rate of T2DM by age, period and median birth cohorts in BRICS,1990 to 2019. Description: The file includes five worksheets: ‘Brazil-Female’, ‘Brazil-Male’, ‘China-Female’, ‘China-Male’, ‘India-Female’, ‘India-Male’, ‘Russian Federation-Female’, ‘Russian Federation-Male’, ‘South Africa-Female’, ‘South Africa-Male’. In each sheet, the data indicate the incidence of T2DM per 100 000 men/women in each specific age group during the period. The diagonal line from the lower left corner to the lower right represents the birth cohort.

## Data Availability

Resources on the incidence of T2DM in each country, as well as national population data, are available from http://ghdx.healthdata.org/gbd-results-tool

## Abbreviations

T2DM: Type 2 diabetes mellitus
BRICS: Brazil, Russia, India, China, South Africa
GBD2019: Global Burden of Disease Study 2019
NCD: Non-communicable disease
YLDs: years lived with disability
APC: Age-Period-Cohort
BMI: Body Mass Index
